# The Global Health Crisis and Our Nation's Research Universities

**DOI:** 10.1371/journal.pntd.0000635

**Published:** 2010-02-23

**Authors:** Sandeep P. Kishore, Gloria Tavera, Peter J. Hotez

**Affiliations:** 1 Weill Cornell/Rockefeller/Sloan-Kettering Tri-Institutional MD-PhD Program, New York, United States of America; 2 Instituto Nacional de Salud Pública, Santa María Ahuacatitlan, Cuernavaca, Morelos, Mexico; 3 Sabin Vaccine Institute and Department of Microbiology, Immunology, and Tropical Medicine, The George Washington University, Washington, D.C., United States of America

On September 14^th^, 2009, the presidents of five United States universities—Boston University, Brown, Duke, Johns Hopkins, and the University of Washington—and representatives of over 50 North American institutions convened for the first meeting of the Consortium of Universities for Global Health (http://www.cugh.org). The meeting was in response to the demonstrated passion and interest of students in the field of global health and the responses needed from universities to cope with increasing student interest in this field. Of 37 institutions surveyed that feature global heath programs, the number of undergraduate and master's level students studying in the field has doubled since 2006. In this arena, growing student movements have helped lead the way. Organizations such as Clinton Global Initiative Universities have also successfully tapped into university student interest in global public health outreach and research. To be sure, universities are well poised to lead such a movement for global health: They are independent organizations, boast central missions to promote public welfare, and possess copious resources and knowledge to share with partner institutions globally [1].

All the while, what remains overlooked in this rapidly expanding global health movement is real innovation for prevention and treatment of the diseases of poverty; existing drugs, some more than 50 years old, accrue microbial resistance and, on the whole, exist only in unadjusted dosages for pediatric patients [2]. What's more, some drugs (e.g., the arsenicals and pentamadine) exhibit toxicities that we would consider unacceptable if they were widely used in the developed world.

The innovation gap for the diseases of poverty is growing at a frightening pace. For instance, some estimates indicate that the total research and development funding for diabetes is more than 15 times that of malaria, and more than 100 times that of other parasitic infections such as hookworm, elephantiasis, and schistosomiasis. Because these diseases almost exclusively afflict the world's poorest people we must look to universities to provide some of the leadership on this issue. Other funding bodies—including the US National Institutes of Health (NIH)—are providing fresh capital for basic science research, but more funds are needed to cope with the global burden of neglected diseases [3]. Certainly, Dr. Francis Collins, the new NIH Director, has affirmed a strong commitment to global health in his strategic vision. but while we feel that he has made almost heroic efforts to ensure prioritization of a global health research agenda, the harsh reality is that the NIH budget has been essentially flat since 2003 [4]. The American Recovery and Reinvestment Act committed to $10 billion of stimulus funds to the NIH, US$8.2 billion of which is to be directed to scientific research priorities [5].

Treating the full range of diseases prominent in the global health arena is important. While we advocate an increase in the overall investment in global health disease research, we specifically call for a new and prominent focus on research for neglected tropical diseases (NTDs), a group of infections that together rival the disease burdens of more widely known global health epidemics, yet receive especially limited research and development [6]. The most recent global estimate of funding on all neglected diseases is at US$3 billion, with nearly three-quarters of the research funds earmarked for HIV/AIDS (40%), tuberculosis (15%), and malaria (18%) [7]. Just 0.4% of this pot (less than $10 million each) is targeted for diseases like leprosy, Buruli ulcer, or trachoma which affects over a billion people, 16% of the global population [7].

## By increasing funding not only for global health research, but also for research in NTDs, universities could make an impact twice as strong

Right now, there are approximately 50 U.S. universities with endowments that exceed US$1 billion, and yet their contribution to global health research, implementation, and training remains relatively meager. For the most part, research and education for the neglected diseases in developing countries are still not a substantial component of most university agendas. At most of our nation's major research universities, internal budgets devoted to global health research are far below the annual salaries of their university presidents and chief executive officers. Despite stated public commitments to global health, many of the university centers with global health arms are still supported with external funds.

So how can research universities further harness student and faculty interest in global health to make a meaningful impact for NTD research?

Here, we propose three specific steps.

## First, universities should develop new seed funds for NTD research

Similar to the call by the United Nations for countries to donate 0.7% of their GDP to help reach the Millennium Development Goals, this would represent a concrete university commitment to neglected disease research, training, and education. The dollar amount of the fund could vary by school, but a useful rubric could be the salary of the university president (for example). The median salary of a private university president is US$627,750, with 23 presidents making more than $1 million [8],[9].

What would these funds accomplish? They would not be enough to seed new laboratories but could supplement existing ones, such as for new graduate student fellowships or fresh interdisciplinary symposia (e.g., in evolutionary microbiology). As an example, the University of Pennsylvania has gone so far as to create interdisciplinary professorships in global health, called “Integrating Knowledge Professors,” with similar funds [5]. The bulk of these funds could be used to fund innovative new US$100,000 projects in the spirit of the Gates Explorations. Alternatively, these funds could provide operational support for neglected diseases centers, like the existing center at the University of California. Berkeley, or finance core facilities that develop and maintain novel transgenic parasite lines.

The start-up funds could also be used to help recruit human resources and laboratory space for product development partnerships. The funds may even help provide fellowship support between universities internationally (e.g., Duke and the National University of Singapore's partnership in dengue research). This collaborative effort may be key in empowering countries to sustainably address their own health epidemics while building capacity for scientific research. Funding could also go toward a subsidy or partial fee waiver for students in certain global health programs who partake in a social service related to neglected diseases—akin to the Global Science Corps (Princeton University; http://sites.ias.edu/sig/gsc). The possibilities are limitless, but with a university seed fund for diseases of poverty a little would go a long way.

## Second, universities should eliminate barriers concerning intellectual property around neglected diseases

It's no secret that current intellectual property schemes hinder both innovation and access to essential medicines for the poor in developing countries [10]. To ensure that licensed innovations remain available for drug development, universities should create a research exemption for neglected diseases, retaining all intellectual property rights for the purpose of neglected disease research.

On November 9^th^, 2009, six universities—Harvard, Yale, Boston University, Oregon Health Sciences University, the University of Pennsylvania, and Brown)—along with the Association of University Technology Managers announced a plan to facilitate access to university innovations with a clause ensuring global access to low-cost products by manufacturers for treatment of infectious diseases [11],[12]. The NIH and the US Centers for Disease Control have also signed on, but many other universities and institutes have not.

Already, several health products (e.g., drugs, vaccines, and diagnostics) for neglected diseases are being developed at University of North Carolina Chapel Hill (African sleeping sickness), University of California (Chagas disease, malaria, and schistosomiasis), Sabin Vaccine Institute-George Washington University (hookworm and schistosomiasis), and Washington University St. Louis (lymphatic filariasis), but care should be taken to ensure that needless intellectual property barriers do not stand in the way of developing new life-saving tools.

The University of British Columbia's new collaboration with iCo Therapeutics to develop and distribute a new low-cost oral formulation of Amphotericin B for visceral leishmaniasis underscores the potential for leadership by universities to push forward alternative (non-financial) metrics for exercising technology transfer [13]. Even baby steps forward—such as providing extra support for talented university technology transfer officers to help develop new therapies for neglected diseases—have immeasurable symbolic and material benefits.

## Third, new metrics for faculty appointments that value neglected disease research should be implemented

The lack of funding, but also the lack of a proper home (professional organizations that provide financial and professional support for academic advancement), for interdisciplinary research is a real issue. Some deans and department chairs show a lack of interest in global health [5] or neglected disease research programs, often in sharp contrast to the interest of their students, trainees, and faculty. The simple rubric of high-impact publications authored and grants received needs to be re-evaluated for this field. The scientific hurdles in the fields of parasitology, for example, where expression systems, mutagenesis, or knockouts can take months (if not years) need to be balanced with comparison to the work of researchers using other model systems. Alternative metrics might, for instance, reward work based on how many quality- and disability-adjusted life years (QALYs and DALYs) are saved [14].

Put frankly, from the perspective of the two graduate student authors (SPK, GT), the current incentives for entering and staying in neglected disease research are few and far between. And what's worse is the danger of our universities (and their researchers) choosing research problems based on their potential commercial value. Devising and developing therapies for the diseases of poverty is not profitable, but the dividends of developing life-saving therapies are priceless. If our universities won't deliver, who will?

University constituents can be prime movers in this field. These commitments will be strengthened only by formal university commitment from the highest levels and unprecedented university collaboration designed to prioritize global public health. At the turn of the 20^th^ century, the presidents of our most prominent universities led the way in reforming medical education in the US and creating the beginnings of today's medical research juggernaut in the developed world. Now, they stand at the cusp of promoting health around the world. Though this is exciting, we question whether real in-house university commitments will now match the fanfare. Will universities put their money where their mouths are?
